# The Multi-Chamber Electronic Nose—An Improved Olfaction Sensor for Mobile Robotics

**DOI:** 10.3390/s110606145

**Published:** 2011-06-07

**Authors:** Javier Gonzalez-Jimenez, Javier G. Monroy, Jose Luis Blanco

**Affiliations:** 1 Department of System Engineering and Automation, University of Malaga, Campus de Teatinos, 29071 Malaga, Spain; E-Mail: jgmonroy@uma.es; 2 Department of Civil Engineering, University of Malaga, Campus de Teatinos, 29071 Malaga, Spain; E-Mail: jlblanco@ctima.uma.es

**Keywords:** electronic nose, mobile robotic olfaction, gas sensing, Metal Oxide Semiconductor Sensor

## Abstract

One of the major disadvantages of the use of Metal Oxide Semiconductor (MOS) technology as a transducer for electronic gas sensing devices (e-noses) is the long recovery period needed after each gas exposure. This severely restricts its usage in applications where the gas concentrations may change rapidly, as in mobile robotic olfaction, where allowing for sensor recovery forces the robot to move at a very low speed, almost incompatible with any practical robot operation. This paper describes the design of a new e-nose which overcomes, to a great extent, such a limitation. The proposed e-nose, called Multi-Chamber Electronic Nose (MCE-nose), comprises several identical sets of MOS sensors accommodated in separate chambers (four in our current prototype), which alternate between sensing and recovery states, providing, as a whole, a device capable of sensing changes in chemical concentrations faster. The utility and performance of the MCE-nose in mobile robotic olfaction is shown through several experiments involving rapid sensing of gas concentration and mobile robot gas mapping.

## 1. Introduction

An electronic nose (e-nose) is a device that detects and recognizes chemical volatile substances using an array of gas sensors, and some kind of signal preprocessing and a pattern recognition algorithm [1]. In the last years, e-noses have generated much interest due to their potential to help in a variety of applications such as food and beverage manufacturing [2], wine brand discrimination [3], fragrance and cosmetics production [4], environmental monitoring [5], medical diagnostics [6] and industrial robotics [7].

The Metal Oxide Semiconductor (MOS) are one of the most popular gas transducers due to their high sensitivity and low price (typically, under 10€ each). They present, however, different shortcomings, among others:

- The need to be pre-heated at temperatures up to 200–500 °C in order to facilitate the interaction with the target gas.
- The acquisition cycle is very long because of their slow response, especially when recovering the baseline level after the exposure to the target gas ends [8]. This baseline level represents the sensor output in absence of target gases and varies with temperature and humidity and among sensors.

Both limitations come from the chemical mechanism underlying MOS sensors, related to the semiconductor behavior when exchanging oxygen molecules between the volatile and the MOS film [9,10]. The first problem, the temperature limitation, is solved by a pre-heating process carried out by built-in heaters which are powered up several minutes before operation. The latter, the long recovery time, is a more serious limitation in applications where we need to take repetitive samples in a short period of time.

**Figure 1.**
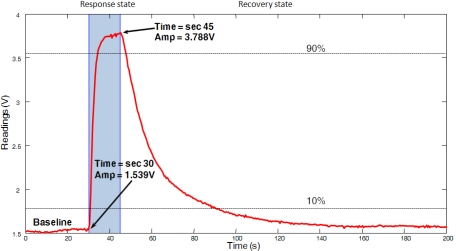
Response of a Figaro TGS 2620 MOS sensor when exposed to a gas source during 15 s (in blue). Observe how the signal response resembles that of two first-order systems: one for the rise phase and another, much slower one, for the decay phase. The excitation signal is unknown, but it is approximate by an ideal pulse of arbitrary amplitude.

Figure 1 shows the response of a typical MOS sensor (a Figaro TGS 2620) when exposed during 15 s at room temperature (approx. 24 °C) to a gas source consisting of a small cup filled with acetone. The measurement was performed in a controlled room where windows and doors were kept closed to avoid airflows as much as possible. It can be seen that the rise takes about 3–4 s, while the decay takes far longer (about 35 s). This sensor response corresponds to an e-nose where the air is aspirated with a small fan. Forced aspirated air is convenient since it speeds up the two processes involved: chemical reactions on the MOS active surface and cleaning of the surface with fresh (non-contaminated) air.

Notice how the sensor response can be properly modeled as a double first-order low-pass filter, with a much higher time constant for the decaying phase. Such a model has been reported and exploited by different authors [11–13].

Particularly, for a mobile robot equipped with smelling capability, which is the main concern of this article, such a long recovery time of the MOS-based e-nose imposes serious limitations to the robot mobility (trajectory, speed, *etc*.), as in the case of [14]. In this paper we propose a new design for MOS-based e-noses which overcomes to some extent this problem. Section 2 relates the important influence of the e-nose slow recovery time in mobile robotic olfaction. The proposed sensor configuration, called Multi-Chamber E-nose (MCE-nose, for short), is introduced in Section 3. Then, its integration into a mobile robotic platform is depicted in Section 4, while Section 5 presents some experiments where it is shown the advantages of the MCE-nose. We end up with some conclusions and discussing future research.

## 2. On the Importance of the Long E-Nose Recovery Time in Mobile Robotic Olfaction

**Figure 2.**
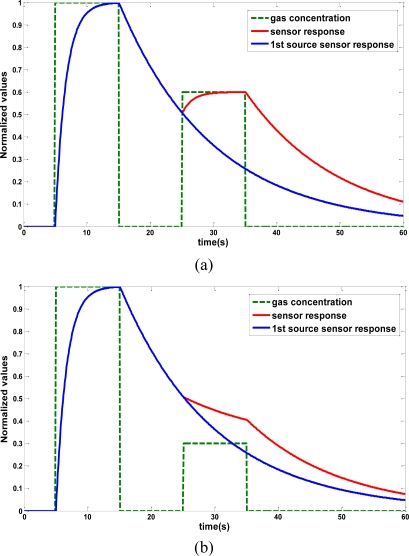

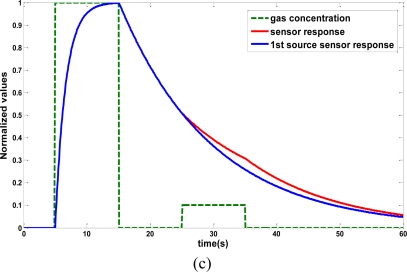
Simulations of the behavior of a MOS sensor when sensing a low gas concentration right after being exposed to a stronger one. The MOS sensor response has been modeled as a two-phase first-order system with time constants 1.7 s, and 14.8 s, for the rise and decay stages respectively (estimated from system identification techniques). Three different scenarios have been simulated varying the source strength ration between both sources: **(a)** 80%, **(b)** 30% and **(c)** 10%. Observe that, when the second gas source is much lower than the first, the response of the MOS sensor (in red) is very similar to that obtained from the first source alone (blue).

For a mobile robot intended to accomplish olfaction-related tasks, the problems associated to the slow recovery of MOS gas sensors are manifested, among others, through the following issues:

- A gas concentration may be masked by another close, stronger one. Suppose we have two gas sources of different concentrations, separated by a short distance. If the robot trajectory first leads to the lower-concentration gas source, both of them will be probably detected. However, if it happens the other way around, the lower one may be overlooked since it could be hidden below the decay of the stronger concentration. Figure 2 displays a simulation of such a scenario.
- Gas concentration maps are not accurate, as a consequence of the integration into the map of unreliable sensed values from the decay phase of the sensor response.
- Gas source search methods that rely on gradient techniques may not be applicable. These methods require to measure and compare the gas concentration at different points, either successive readings (process called as klinotaxis [15]), or simultaneously sensed intensities from two or more sensors (called tropotaxis [15]). For the first case, we cannot trust in the sensor measurement if it is still in the decay phase of the previous sensing.

Thus far, mobile olfaction tasks have managed this limitation in, basically, two ways:

- Slowing down the robot speed to a few cm/s in order to allow the sensor response to slowly follow the gas distribution even in the decaying phases [16].
- Defining paths that force the robot to pass several times over the same locations but along different directions, in such a way that the decay effect is averaged out over all the measurements. This is a common strategy employed to explore a space with the intention of building a gas concentration map, such as in [14].

Clearly, this type of solutions affect the overall efficiency of the olfactory task and, in many cases, it may be even unacceptable for the robot mission. It is important to remark that, for most real robot applications, smell is not by itself the ultimate goal for the robot, but just another of the robot’s senses to gather useful information from the environment (along with vision, range sensing, touch, *etc*.).

## 3. The MCE-Nose

It is well known that wine testers have a very developed and well trained sense of smell. In a typical session, to avoid mixing the smells or tastes of different wine samples, they have to clean their mouths and noses by eating a little piece of bread and wiping their noses, for example. Thus, they undergo a “purge/clean” stage between tests and they also stop for a few seconds to ensure their noses are ready to provide new accurate olfactory information. MOS gas sensors behave in a quite similar way, as they require a time (decay phase) to ensure their readings are accurate.

The MCE-nose proposed here pretends to work in a similar way that wine testers, but taking advantage of the reproducibility of electronic devices to avoid the down-time between readings. Thus, the key idea behind the proposed design is to ignore the MOS sensor output when the decay phase is detected and delegate the sensing task to another clean, almost identical sensor. In order to achieve that, we accommodate a set of redundant sensors in different chambers, which are alternatively activated. Thus, the output signal of the whole setup results from the concatenation of the rise phases of a sequence of MOS sensors.

The design of the MCE-nose aims at providing the following characteristics:

- To shorten the cycle of effective sensing as exposed above.
- To recognize a variety of odors by hosting MOS sensors with different selectivity in each chamber.
- To reduce the influence of residuals from previous measurements by scaling down both the chamber room where the sensors are accommodated and the air circuit volume.
- To speed up the interchange of molecules onto the MOS film by feeding a pressured air flow into the chamber by means of a pneumatic pump.

Next, the three main aspects of the MCE-nose design are exposed: mechanics, electronics, and software.

### 3.1. Mechanical Design

**Figure 3.**
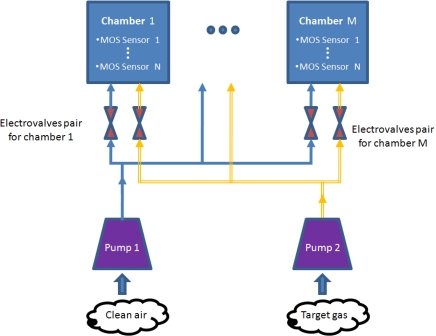
A functional schematic diagram of the MCE-nose. There are two pumps: one aspirating clean air and the other the target gas. At each time, only one chamber is receiving the target gas while the other M-1 chambers are being purged with clean air.

Figure 3 shows a schematic diagram illustrating the interconnections of the different components of the proposed e-nose. The design is conceived to comprise a general number of *M* chambers with *N* MOS sensors each. All chambers are identical and contain the same set of sensors. Chambers are also isolated from each other, that is, no airflow circulates between them.

There are two pneumatic circuits: one for clean air and one for the target gas (*i.e*., odor charged), which are connected to each chamber. Clean and contaminated air flows are taken from opposite sides of the MCE-nose device via two separate pumps. Besides, clean air is forced to flow through an active carbon filter to eliminate possible impurities.

At any given time, only one chamber is fed with the target gas, while the others *M-1* are being cleaned. This is done thanks to a set of electro-valves placed at the entrance of each chamber, controlled by embedded software built in the MCE-nose micro-controller, as will be described later in this section.

At any time, each chamber can be found in one of following three states:

- *Clean*: A chamber is said to be “clean” if all of its MOS sensors are at their baseline level. This may happen because either the chamber has not being used yet for sensing or because it has been injected with clean air long enough.
- *On-Cleaning*: Opposite to a clean chamber, an on-cleaning one is that whose sensors are not completely cleaned (*i.e*., they have not reached the baseline yet), despite the chamber is being injected with clean air.
- *Active*: The chamber is being injected with the target air. The readings of this chamber are used as the MCE-nose output. As the active chamber changes with time, the MCE-nose output results from the concatenation of the different active chamber readings along time.

**Figure 4.**
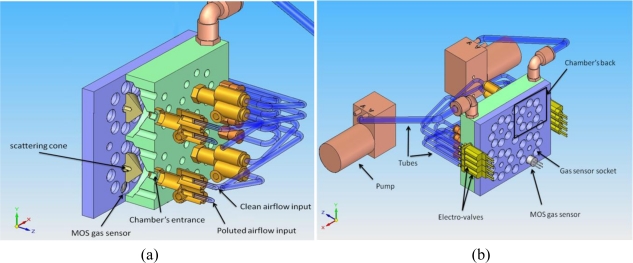
Different views of the 3D model **(a)** upper view, **(b)** bottom view, of the pneumatic circuit and the main block containing four chambers which can accommodate up to 8 MOS sensors each.

Figure 4 shows some of the 3D models created for the current prototype, which consists of four chambers with identical configuration which can accommodate up to 8 MOS sensors each. Our choice of such particular number of chambers obeys to a trade-off between two issues:

- On the one hand, the obvious higher cost and complexity of the device as this number increases: more sensors, valves, A/D converters, *etc*. as well as problems for dissipating heat on the PCB, power consumption, *etc*.
- On the other hand, the possibility of having an array of sensors at the baseline level and, consequently, the possibility of sensing at a higher frequency.

**Figure 5.**
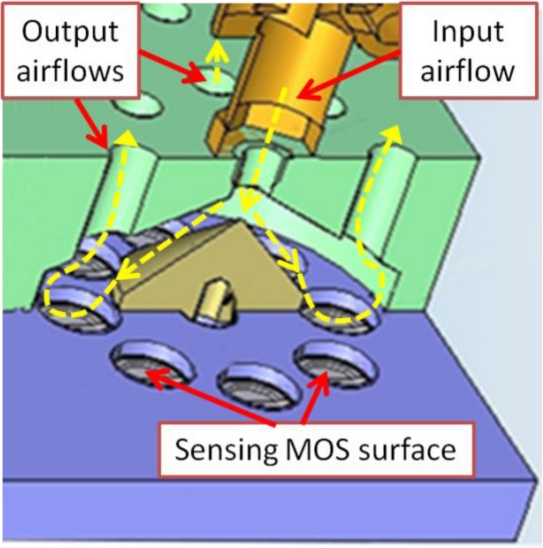
Approximate airflow scheme inside each chamber.

The main block, which accommodates the four chambers, has been fabricated of resin with a stereolithography machine. Each chamber has a circular array of eight sockets to lodge MOS sensors of standard size (8 mm diameter). It can be appreciated in Figure 4(b) how the sensors are placed. They are introduced from the bottom side of the main block, leaving the sensing surface inside the chamber and, at same time, facilitating the electronic connections (pin soldering). A cone at the entrance of the chamber scatters the incoming airflow evenly directing it towards the active sensing surface of the sensors. The air is then forced to escape through the upper orifices of the chamber, as illustrated in Figure 5.

Each of the eight sockets can lodge a different sensor. In our case, each chamber contains seven different MOS sensor, with the extra socket employed for a temperature sensor (ADT7301). The seven MOS sensors were selected with different gas selectivity in order to facilitate odor classification. This amount of sensors has demonstrated to be large enough to allow the recognition of a wide range of odors.

**Figure 6.**
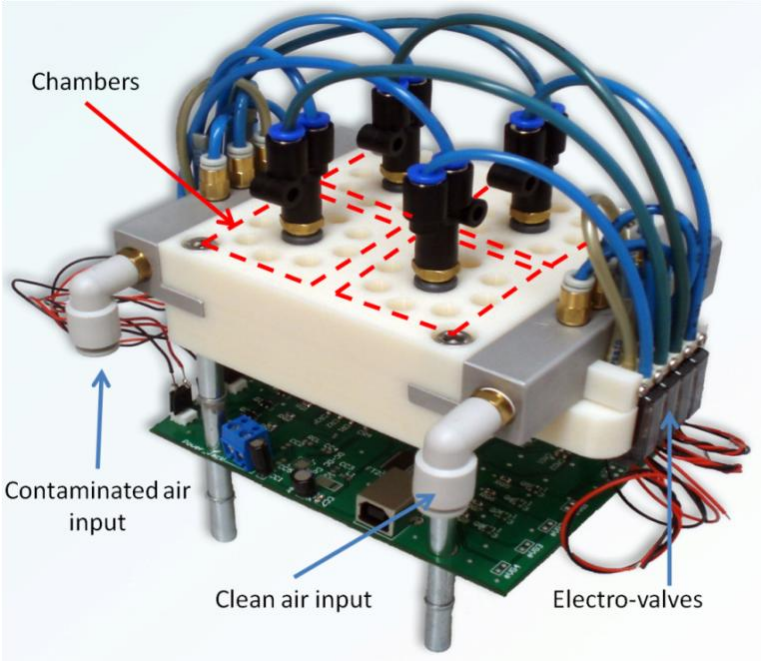
The complete MCE-nose. The current prototype contains four chambers, hosting eight different MOS sensors each.

In our prototype, the pumps mounted are EAD NEO IP3 diaphragm pumps: 15 V dc, 180 kPa maximum attainable pressure, and working flow of 4 Lpm. For each of the chambers, two SMC S070C6BG32 electro-valves are used: one for the clean and one for the polluted air flow. To interconnect pumps, electro-valves and chambers, we have used standard pneumatic PVC tubes with diameters of 8 and 3 mm, as well as the required plugs. Figure 6 shows a picture of the built prototype.

### 3.2. Electronics

**Figure 7.**
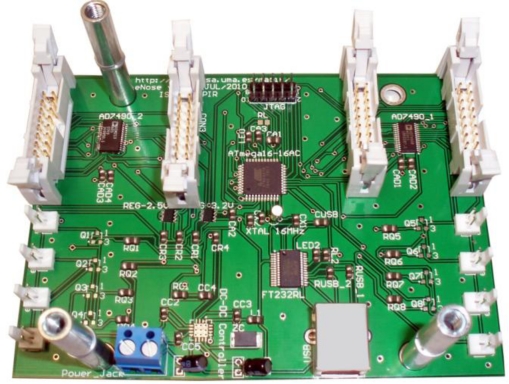
PCB where all the electronic components have been mounted.

Any conventional MOS-based e-nose requires a minimum of electronics to cope with sensor pre-heating and sensor readings, including signal conditioning and A/D conversion. In our design, the electronic module has to take care also of the synchronization of the pneumatic circuits by controlling the eight electro-valves (one pair for each chamber). As seen in Figure 7, such electronics has been mounted on a single printed circuit board (PCB) which is connected to all the components by means of four 16-pin connectors (for the gas and temperature sensors) and eight 2-pin connectors (for the electro-valves).

The core component of the PCB is an ATMega16 8-bit microcontroller at 16 MHz, which provides 32 programmable I/O lines to control two A/D 16-channel 12-bit converters (connected to the gas sensors), four temperature chips (placed inside each chamber to measure working temperature), and the eight electro-valves. Additionally, the PCB comprises a USB connection to a PC host for easy interfacing and a standard JTAG interface for development.

### 3.3. Embedded Software

**Figure 8.**
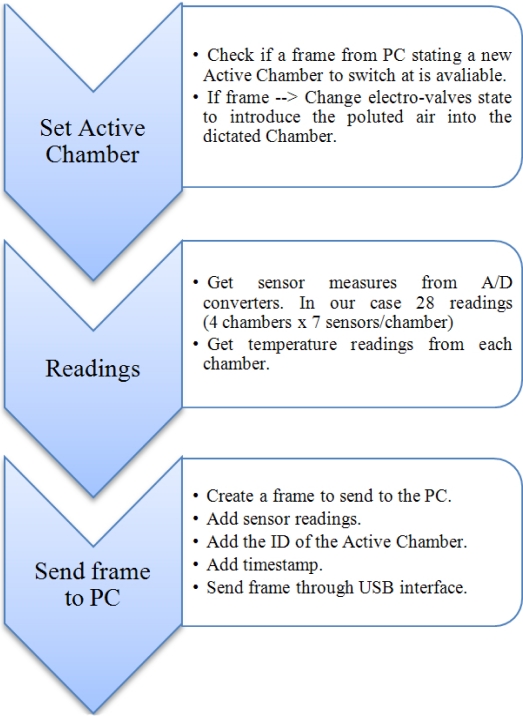
Operation flow of the embedded software.

The firmware we designed for the ATMega16 microcontroller is in charge of controlling the behavior of the MCE-nose components. The operation flow is based on three main stages, described in Figure 8.

- The first stage checks if a data frame containing the information about the next active chamber is received from the PC. If this is the case, the appropriate signals are issued such as the electro-valves switch the airflow into the newly selected active chamber. Notice that the switch strategy that dictates the active chamber at any given time has not been embedded into the microcontroller, but it relies on orders from the computer. This decision obeys to our interest in implementing high-level switching strategies that may take into account information from other sensors and the robot task.
- The second stage is in charge of collecting the readings from all the sensors of the MCE-nose (28 MOS and 4 temperature sensors in our case). This is done by means of two A/D 12-bits converters of 16 channels each.
- Finally, all the collected data are packed into one frame, which is assigned a timestamp and the ID of the active chamber. This data frame is then sent to the PC via a USB-to-serial UART interface (FT232RL).

### 3.4. Calibration of Gas Sensors

As depicted above, the output signal of the MCE-nose results from the concatenation of the rise phases of identical MOS sensors, placed in the different chambers. Nevertheless, in practice, such identical sensors do not respond the same and thus, a calibration is required in order to make their responses as similar as possible. For such calibration, we have to compare the readings of all chambers when exposed to the same concentration.

**Figure 9.**
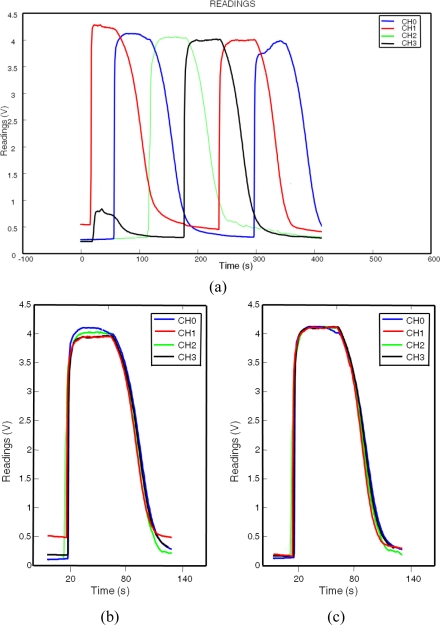
**(a)** Readings of four TGS-2602 sensors placed in each chamber of the MCE-nose prototype during the calibration procedure. **(b)** Comparison of the four sensor readings before calibration, and **(c)** after it.

To ensure that all chambers are flooded with the same gas concentration, the four chambers where individually and sequentially flooded during 60 s, allowing their sensors to reach the steady state (see Figure 9).

Since only the baseline and the rise phase of each sensor are of interest for the MCE-nose output (as the decay phases are discarded), we compensate outputs of sensors in chamber 1, 2 and 3 to achieve the baseline level and the amplitude of the reference output (chamber 0). Concretely:

- At the beginning of each experiment, the differences in the sensors baseline were compensated by adding an offset to each sensor. Under the assumption of short time experiments (as in our case), the baseline drift due to humidity, temperature or even poisoning [8] is negligible and, therefore, has not been taken into account.
- A multiplying factor was estimated for each sensor to ensure identical gain. To account for the non-linear behavior of the sensors we selected an average gain computed from three different concentrations.

Figure 9(a) shows the readings of TGS-2602 sensors placed in each chamber of the MCE-nose prototype during the calibration procedure. It may be notice that even before calibration the readings of the four sensors are all very similar (as reasonably expected). Figure 9(c) plots the readings of the same sensors after the calibration has been carried out.

## 4. Integration of the MCE-Nose into a Mobile Platform

**Figure 10.**
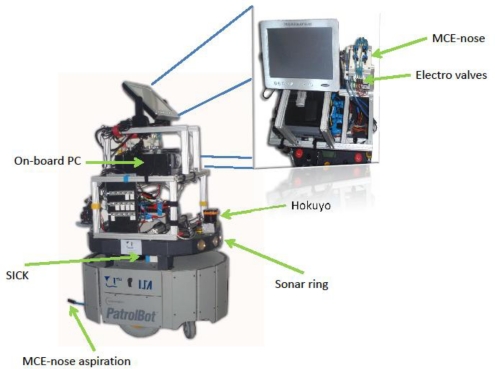
The MCE-nose integrated in a mobile platform PATROLBOT mobile base.

The MCE-nose presented in this paper has been designed to be integrated into a mobile robot. Figure 10 shows a PatrolBot mobile platform [17] with the MCE-nose already integrated into it. The robot is also equipped with a SICK and a Hokuyo laser range scanners and a sonar ring to provide the necessary functionality for localization and obstacle detection.

One of the main advantages of the MCE-nose is its suitability for mobile olfaction tasks. The mechanical design of the MCE-noise opens a variety of possible configurations:

- It can work either as a MCE-nose (as explained in the previous section) or as a conventional e-nose by using only one of the chambers. This may be convenient in some phases of an olfaction task (e.g., odor classification).
- Since the aspiration is carried out through a tube, the air input can be conveniently placed at any point around the robot. This allows the MCE-nose to be mounted at any place on the platform, no matter its shape or size. Also, olfaction strategies that need to compare concentrations from several points around the robot (gradient techniques) are easily accomplished by just moving the aspiration tube, for example, with a servo motor. Even if no comparison is needed, having such capability bears some advantages: (1) we are not limited by the robot nonholonomic constraints while sampling the workspace (*i.e.*, the robot is not allowed to move in any direction at any time), and (2) we reduce the air disturbance caused by the robot movement to a minimum, since we reach the target point with the tube which generates a negligible turbulent airflow.

Considering the possibilities offered by a MCE-nose integrated into a robotic platform, it is necessary to account for high level software able to exploit such potential for any robotic olfaction task. These possibilities include: switching between chambers, focusing only on some specific (more suitable) MOS sensors from the array, taking into account the robot mobility as well as surrounding information from other sensors of the robot (laser scanner, sonar, …), *etc*.

**Figure 11.**
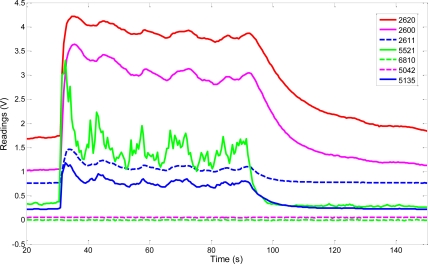
Readings from seven different MOS sensors within a chamber when exposed to acetone.

Among others, this software has to deal with the following tasks:

- To detect abnormal levels of a gas (probably while accomplishing a non-specific olfaction mission), through a pilot “watchdog” sensor from the MCE-nose. This could be done instead, by a static gas sensor network deployed in the environment.
- To classify the target gas. MOS sensors have low selectivity, so the multivariate response of an array of chemical gas sensors with broad and partially overlapping selectivity can be used as an “electronic fingerprint” to characterize a wide range of odors or volatile compounds by pattern-recognition means [18]. For this task, typically only one chamber is necessary, thus no chamber switch is required. As an illustrative example, Figure 11 shows the responses to a specific odor of seven different MOS sensors within one chamber.
- Measuring the target gas concentration is crucial for almost all robotic olfaction tasks, including gas source localization and gas mapping. With the purpose of obtaining the best estimation of such concentration, is advisable to select, from the sensors of each chamber, those more sensitive to the target gas. Referring to Figure 11, sensors S2620 and S2600 are good candidates for gas concentration purposes due to their high sensitivity to that gas.
- To control and manage complex switch strategies which could take into account not just the gas sensor readings, but also information provided by other sensors (laser scanner o camera), as well as the olfaction task at hand (e.g., plume detection, gradient following, *etc*.).

Such software has been implemented under the Open Mobile Robot Architecture (OpenMORA) [19], based on MOOS [20] and MRPT [21]. This architecture allows us to easily control a robot platform and the available sensors as range lasers, cameras or sonar, as well as providing high level functionality as obstacle avoidance, autonomous path planning or localization.

## 5. Experiments with the MCE-Nose

This section describes different experiments we have carried out to validate the MCE-nose with regard to the improvement in rapid sensing of gas concentrations. The experiments consist of a static smell test, a mobile experiment with multiple gas sources, a mobile test with different gas concentration sources and finally a gas mapping experiment. Since the kind of gas to sense was known a priory, neither odor classification nor sensor selection was required here. The implemented switch strategy is based on two rules for deciding when to switch and what chamber to switch to:

- Rule 1: A switch of chamber must happen whenever the sensor readings from the current active chamber (being fed with the input stream) start to decay.
- Rule 2: Provided a switching event has been triggered by rule 1, it is necessary to check the state and sensor levels of all the *M* chambers (*clean*, *on-cleaning* and the *active* one). The one with the lowest sensor readings is chosen to be the next chamber to commute to.

### 5.1. Static Test

**Figure 12.**
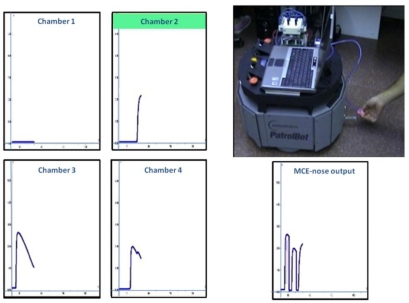
Snapshot of the MCE-nose static smelling experiment. The four plots on the left side present the readings of each of the four chambers of our current prototype, while the MCE-nose output is shown on the bottom-right plot. The active chamber is marked in green (chamber 2 in this case).

In this experiment the robot was kept still, being the gas source (a small cup filled with acetone) the mobile element. The experiment consisted in repeatedly presenting the gas source to the MCE-nose air input, waiting a few seconds and moving it away. Figure 12 shows a snapshot of the experiment and the responses obtained with every chamber (conventional e-nose) and with the MCE-nose (the concatenation of the active chamber readings over time). It can be appreciated how the MCE-nose output is able to capture the (three) different exposures by changing to a clean chamber whenever the response of the active one (being odor flooded) starts decaying.

### 5.2. Detecting Multiple Odor Sources

**Figure 13.**
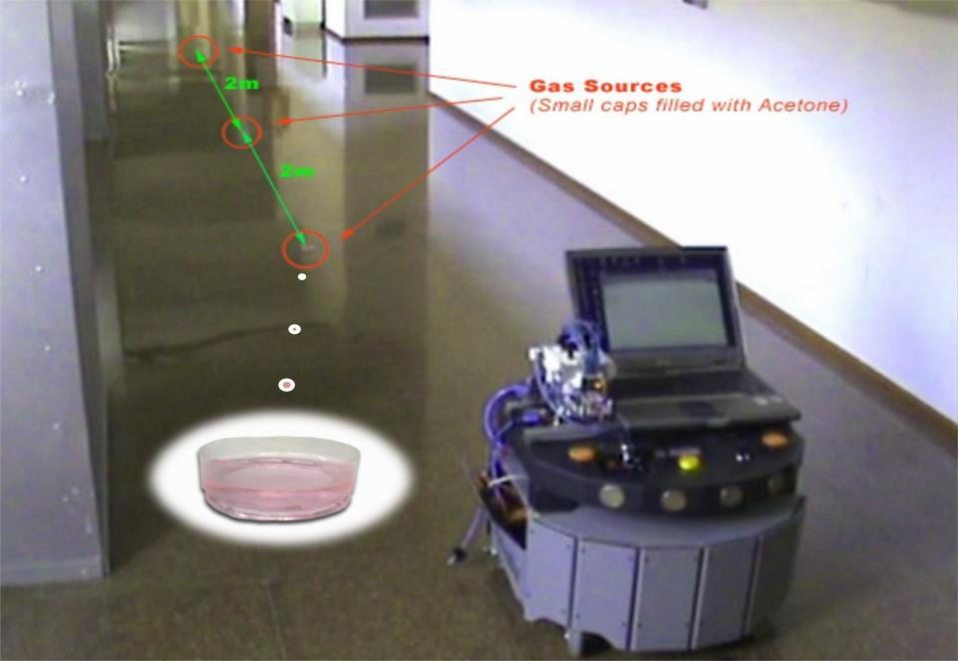
Description of the multiple gas source experiment. Three small cups filled with acetone where placed along the robot trajectory to test the behavior of the MCE-nose.

**Figure 14.**
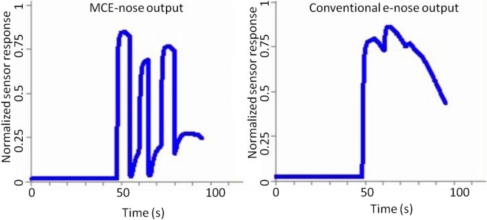
Output readings comparison between a conventional e-nose (right) and the MCE-nose (left) for the multiple gas source experiment. It can be appreciated how the MCE-nose can clearly distinguish the three gas sources, while a conventional e-nose can hardly detect the second source, while the third one became completely unnoticed.

The second experiment was designed to test the behavior of the MCE-nose in the case of multiple gas sources in a more realistic robotic scenario. The scenario consists of a long corridor where three equal-sized small cups filled with acetone were placed at 2 meters from each other. Figure 13 displays the experiment setup, and a picture of the MCE-nose integrated in the PatrolBot platform. For the experiment the PatrolBot was commanded to move in a straight line at a constant speed of 20 cm/s. Figure 14 illustrates the comparison between the outputs of a conventional e-nose (one chamber) and the MCE-nose.

Notice that for such a robot speed, the readings provided for a conventional e-nose do not reveal the presence of the three odor sources and the low concentration zones between gas sources are not correctly gauged. The common solution to this problem would be to slow down the robot speed, so the MOS sensors could have time to recover their baseline level, which is not possible or practical in many real robotic applications. Observe, on the other hand, that the MCE-nose is able to provide more accurate measures.

Nevertheless, differences in the peak amplitudes between the MCE-nose and the conventional e-nose can be appreciated, as well as differences among the amplitudes of the different “equal-sized sources” in both cases. These differences are probably due to non controlled physical conditions of the environment, where small turbulences dominate the gas dispersion, making almost impossible to exactly reproduce the same experiment for different sources and runs.

### 5.3. Detecting Multiple Odor Sources of Different Concentrations

The objective of this experiment is to demonstrate that using the MCE-nose, the problem of disguising lower concentrations or even additional gas sources (as stated in Section 2), can be notably palliated.

**Figure 15.**
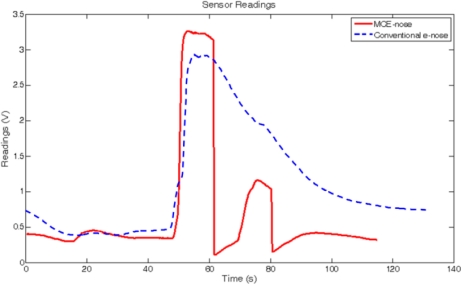
2D comparison of the raw readings between a conventional e-nose (dashed blue) and the MCE-nose (solid red), when faced to two sources of different concentration.

**Figure 16.**
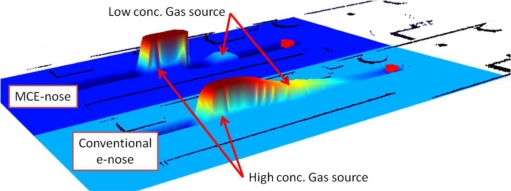
3D comparison of the ICP SLAM generated maps between a conventional e-nose and the MCE-nose when faced to two gas sources of different concentration.

The experiment was carried out in the same scenario as the previous experiment. In this case, only two gas sources separated one from each other 2 m were used. The first one was a wide open vessel (approximately 15 cm diameter), while the second one was a small (4 cm diameter) cup covered by a grid lid to reduce the gas dissipation. Using this setup, two gas sources of different concentrations were presented to the robot along its path. Figure 15 shows the raw readings of the experiment. These values (after normalization) along with the robot position estimated by an ICP-based SLAM process give rise to the map shown in Figure 16. The ICP-based SLAM method is a non-probabilistic approach to simultaneously computing the robot position and building the map from the 2D laser scans gathered by the robot [22].

We must remark the improvement in the detection of a low concentration source after a high one. From a comparison of the “peak” concentrations from the MCE-nose and the conventional e-nose in Figure 16, one may wonder why in the former case the peak seems to extend in a larger area. However, observing the raw readings in Figure 15, it becomes clear that the chamber’s switch in the MCE-nose takes place as soon as the decay phase starts. Thus, the observed differences are only due to the real differences between experiment repetitions. The MCE-nose switches to a different chamber when the readings from the active chamber present a relative decay greater that a given threshold. This threshold was set to 0.1 volts in the current experiment (that means that the readings of the active chamber must decay at least 0.1 v before switching) to avoid miss-switches due to noise or spurious readings. Decreasing the threshold value would mean faster switching after a gas source is detected, but it could then produce non-desired switches due to noise, spurious or because of the small fluctuations inherent in MOS sensors.

### 5.4. Gas Distribution Mapping

**Figure 17.**
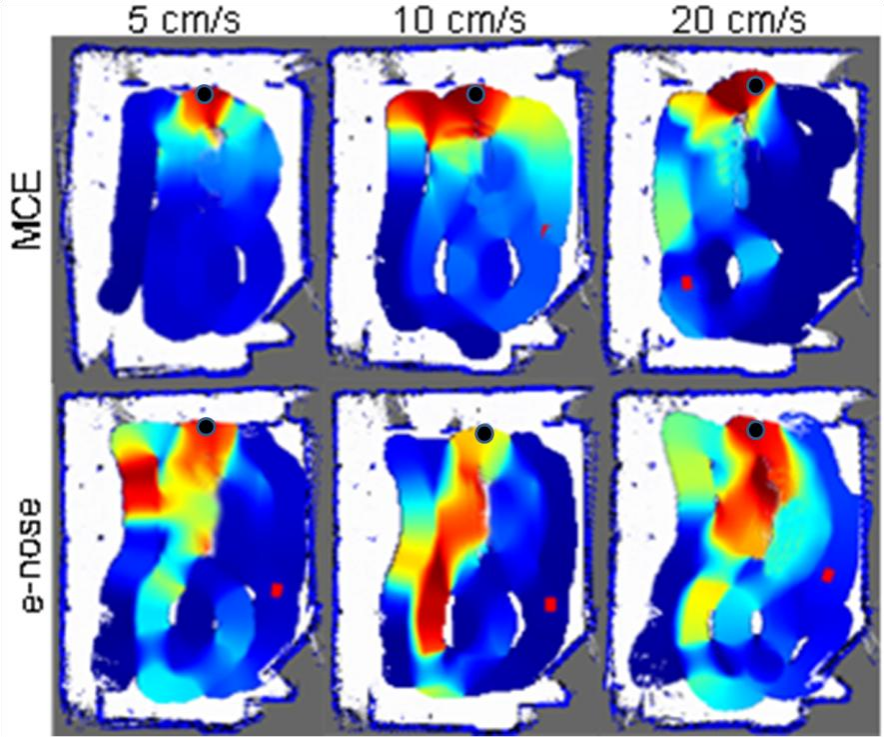
A comparison of the gas concentration maps produced by a conventional e-nose and the MCE-nose for three different robot speeds.

The objective pursued with this experiment was to analyze the performance of the MCE-nose when creating a gas distribution map (a map of relatively measurements of the gas concentration) of a room. A gas source composed by a 10 × 2 cm container filled with acetone was placed in a 6 × 4 meters empty room, next to a wall (marked as a black dot in Figure 17). The robot was commanded to move following a predefined set of way-points to force the MCE-nose to prove most of the space.

To be able to compare the results obtained in different trials, a methodology was established to ensure similar conditions in the room. Door and windows were kept closed during the experiments and sensors were conveniently preheated before operation. After each trial, the room was purged of residual gases by opening the door and windows, creating a strong airflow of clean air for at least 5 min.

Figure 17 shows a comparative between the MCE-nose and a conventional e-nose for three different robot speeds. Each map represents the gas distribution estimated in the room at the end of the robot trajectory, making use of the robot positions given by an ICP-based SLAM method and the Kernel DM+V algorithm [23]. It is important to keep in mind that these maps come from different runs of the experiment and, even though we have tried to reproduce the tests in the same conditions, it is inevitable the appearance of some gas patches from one test to another. In our opinion, this explains, for example, the high concentrations near the source when using the MCE-nose at 10 cm/s.

In spite of this consideration, it can be seen how the MCE-nose is able to localize the gas source more accurately than a conventional e-nose. This improvement is more apparent when the robot speed is increasing, as the slow recovery effect of the sensor will no longer allow the sensor response to follow the gas distribution, increasing in the case of a conventional e-nose the dispersion along the path of the robot. This allows the MCE-nose to perform a simple gas reconnaissance of the environment in a shorter time while obtaining higher-quality results.

## 6. Conclusions and Future Work

In this work, we have presented a new electronic nose to deal with the problem of the long recovery period of MOS gas sensors. This is a serious drawback for mobile robot olfaction since a rapid measurement cycle is required in many olfaction-related tasks: source finding, gas concentration mapping, *etc*.

The MCE-nose presented in this paper partially overcomes this problem by accommodating a set of redundant sensors in different chambers, which are alternatively activated, ignoring the sensor output when a decay phase is detected and delegating the sensing task to another clean, almost identical sensor. The output signal of the whole setup results then from the concatenation of the rise phases of a sequence of MOS sensors.

A prototype of the MCE-nose has been built and integrated in a mobile robotic platform under the OpenMORA robotic architecture. It has been tested in different scenarios showing an important improvement when sensing rapid gas concentration fluctuations or multiple odor sources, as well as a notable increment in the accuracy of gas source localization when generating gas concentration maps, even in the case of increasing the robot speed several times.

Future work includes some improvements in the MCE-nose, such as the incorporation of another electro-valve to purge the pneumatic circuit or the enlargement of the tubes section to increase the airflow through the sensor’s surface. Obviously, we are also very interested in the exploitation of the presented MCE-nose to real, out-of-the-lab applications.
